# Vaginal Clinical Isolates of *Candida albicans* Differentially Modulate Complosome Activation in Vaginal Epithelial Cells

**DOI:** 10.3390/jof11070501

**Published:** 2025-07-03

**Authors:** Samyr Kenno, Natalia Pedretti, Luca Spaggiari, Andrea Ardizzoni, Manola Comar, Wilfried Posch, Robert Treyde Wheeler, Samuele Peppoloni, Eva Pericolini

**Affiliations:** 1 Department of Surgical, Medical, Dental and Morphological Sciences with Interest in Transplant, Oncological and Regenerative Medicine, University of Modena and Reggio Emilia, 41124 Modena, Italy; andrea.ardizzoni@unimore.it (A.A.); samuele.peppoloni@unimore.it (S.P.); eva.pericolini@unimore.it (E.P.); 2 Department of Clinical, Medical, Surgical and Health Sciences, University of Trieste, 34100 Trieste, Italy; natalia.pedretti@units.it (N.P.); manola.comar@burlo.trieste.it (M.C.); 3 Clinical and Experimental Medicine PhD Program, University of Modena and Reggio Emilia, 41124 Modena, Italy; luca.spaggiari@unimore.it; 4 Institute of Hygiene and Medical Microbiology, Medizinische Universität Innsbruck, 6020 Innsbruck, Austria; wilfried.posch@i-med.ac.at; 5 Department of Molecular and Biomedical Sciences, University of Maine, Orono, ME 04473, USA; robert.wheeler1@maine.edu; 6 Graduate School of Biomedical Sciences and Engineering, University of Maine, Orono, ME 04473, USA

**Keywords:** complement, complosome, vulvovaginal candidiasis, *Candida albicans*, vaginal epithelial cells

## Abstract

The complosome controls different activities in innate immune cells and epithelial cells; however, its role in the response of VECs to *Candida* remains untested. In this in vitro study, we compared two clinical vaginal strains of *C. albicans*, namely, a Colonizing strain from a healthy woman and a strain from a patient with vulvovaginal candidiasis (VVC), for their ability to activate the complosome and release anaphylatoxins in vaginal epithelial cells (VECs). Our results show the following: (i) both strains triggered the cleavage of C3 into C3a and C3b within VECs, while infection with the Colonizing strain led to greater release of the anaphylatoxin C3a; (ii) infection with the VVC isolate led to a strong reduction in both C5 and C5a in VECs, while no increase in C5a release was observed after infection with either strain; (iii) cathepsin-family gene expression and cathepsin D activity were reduced in VECs infected with the VVC strain but not in those infected with the Colonizing strain; (iv) infection with the Colonizing strain induced a significant increase in intracellular C5aR1 while intracellular C3aR levels remained unchanged. Collectively, our data suggests the propensity of this VVC strain to inactivate the C5/C5aR1 axis and to reduce the C3/C3aR axis, dampening the activity of the complosome in VECs. These effects exerted by the VVC strain suggest a novel strategy of immune evasion by *C. albicans* and may open new perspectives for finding new therapeutic targets against vaginal fungal infections.

## 1. Introduction

Vulvovaginal candidiasis (VVC) is an infection that affects approximately 75% of women worldwide, with no specific predisposing factors. Under certain specific circumstances, around 5–8% of women with VVC may experience recurrent forms (RVVC), consisting in 3–5 episodes of VVC per year [1]. *C. albicans* is part of the human microbiota, and it normally dwells in the oral, gastrointestinal and genital tracts [2]. VVC is predominantly caused by *C. albicans*, which plays a primary role in the development of the disease. However, non-*albicans Candida* species, such as *C. parapsilosis*, *C. glabrata*, *C. tropicalis* and *C. krusei*, also trigger the disease [3]. Symptoms associated with VVC include vulvar itching, swelling or redness, but they may progress to vulvar edema, fissures, excoriations or thick, lumpy discharge. Symptoms cause stress and discomfort in affected women, influencing both their personal relationships and work life [4].

A different immune response occurs in symptomatic patients with VVC compared to asymptomatic women, namely, increased neutrophilic infiltration with more fungal hyphae detectable in the vaginal swabs and increased exposure of β-glucan on such hyphal fragments [5]. This suggests that the immune response may polarize differently when the vaginal epithelium is in contact with pathogenic VVC-associated *C. albicans* strains versus Colonizing *C. albicans* strains [6]. By using an in vitro model including a monolayer of VECs, routinely used to mimic vaginal mucosa [6], our studies revealed a stronger tendency to induce fungal shedding and epithelial cell exfoliation in VVC-associated *C. albicans* strains compared to Colonizing *C. albicans* strains. Interestingly, our data also suggested that a selected Colonizing *C. albicans* strain (Ca 14314) differentially activates integrins, ferroptosis and type I interferon pathways in comparison with the VVC *C. albicans* strain (Ca 01887) [6]. However, the major mechanisms behind the differential responses in VECs induced by VVC-associated or Colonizing strains remain elusive.

The complement system is known to mediate immune responses to disseminated candidiasis, but its role in VVC onset has not been explored in depth. It has been recently demonstrated that following the estrogen-induced overexpression of the fungal cell surface protein Gpd2, the complement regulatory protein Factor H accumulates on the surface of fungal cells [7]. These results are in line with work showing that *C. albicans* can inhibit the complement system and its downstream effector functions by exposing FH-binding molecules (such as the moonlighting protein Hgt1) or by releasing Pra1 [8,9,10,11,12].

The complement system is a complex network of more than 30 proteins that can induce the killing of microorganisms by the formation of the terminal complement complex [11]. The activation of the complement system occurs via three main pathways: classical, lectin and alternative [13,14,15]. These three pathways have a common purpose, which is the induction of C3 and C5 cleavage into the powerful anaphylatoxins C3a and C5a [16,17]. This cleavage is believed to take place largely in the extracellular space, and the generated anaphylatoxins play pivotal roles in activating inflammation and the acute immune response. Moreover, complement activation leads to the induction of the opsonin C3b and downstream products (C5b, C6 and C9). The latter form the membrane attack complex (MAC), which ultimately leads to microbial lysis [17,18]. Since the fungal cells are protected from the lysis by MAC thanks to the composition of their cell wall, C5, C3a and C3b become the main complement effectors in the context of antifungal defenses. The idea that these complement components play a pivotal role in antifungal defenses is strengthened by the increased susceptibility to systemic fungal infections in C3- and C5-deficient mice [19]. In addition, it has been demonstrated that during systemic candidiasis, an efficient antifungal response is provided by C5a through the engagement of C5aR1 receptors [19].

Complement proteins are mainly produced in the liver, but they can also be secreted by circulating monocytes, macrophages and neutrophils, as well as in tissues by endothelial cells, airway epithelial cells and fibroblasts [20,21,22,23], although in lesser amounts compared to hepatic production [14]. Recent work has highlighted the role of intracellular activation of the complement components C3 and C5 in the “complosome” [20,21,24]. The complosome has been shown to regulate cellular activities such as mitochondrial activity, glycolysis and oxidative phosphorylation, which orchestrate and support cellular vitality [20]. Dectin-1, a CARD9-coupled C-type lectin receptor that recognizes *C. albicans*, has been demonstrated to trigger intrinsic C5a production in macrophages and to synergize with this complement component in mediating fungal killing [25]. In addition, the activation of intracellular C3aR and C5aR1 (located on mitochondria) by C3a and C5a complement fragments has been demonstrated to increase the levels of reactive oxygen species (ROS), thus leading to the activation of the Nlrp3 inflammasome [26]. The C5a/C5aR1 axis can also modulate the immune response, promoting a more pronounced pro-inflammatory tendency in cells such as macrophages and making these cells active against pathogens [19,27,28].

In contrast to the established role of complement in systemic antifungal responses, the interplay between complement and fungi at mucosal surfaces is not completely understood. However, it has been shown that lung epithelial cell-derived C3 provides protection from lung injury during bacterial pneumoniae [29]. In this context, we analyzed the ability of *C. albicans* to activate the complosome in vaginal epithelial cells (VECs) and to respond differentially to infection with either a VVC or Colonizing *C. albicans* strain. Here, we demonstrate that complosome activation, anaphylatoxin production and the presence of anaphylatoxin receptors occur in VECs, in the context of an in vitro vulvovaginitis model. Overall, these events suggest that *C. albicans* is responsible for complement activation, for C3a and C3b extracellular secretion and for cell-associated C5a production by the vaginal epithelium. Our data suggest that, following their activation, such complement components may directly activate epithelial cells through the anaphylatoxin receptors C3aR and C5aR1.

## 2. Materials and Methods

### 2.1. Fungal Strains and Cell Culture Conditions

The Colonizing strain of *C. albicans* (Ca 14314) and the VVC-associated strain of *C. albicans* (Ca 01887), referred to throughout the manuscript as the Colonizing and VVC strains, respectively, were isolated from vaginal swabs of a healthy colonized woman and a symptomatic woman with VCC, as previously described [5]. These strains are already part of our *C. albicans* strain library. The isolated strains were kept in frozen stocks at −80 °C, defrosted every month and maintained by a weekly passage onto Sabouraud Dextrose Agar (SDA) plates (Oxoid, Milan, Italy). Before each experiment, Colonizing and VVC strains were inoculated in Yeast Extract (1%)–Peptone (2%)–Dextrose (2%) (YPD) broth (Condalab, Madrid, Spain) and incubated overnight at 37 °C under agitation.

### 2.2. Vaginal Epithelial Cells (VECs)

The human A-431 cell line, from vaginal epithelial squamous cell carcinoma (ATCC CLR-1555™), was selected because it is routinely used to represent the vaginal epithelium [30]. The A-431 vaginal epithelial cells (VECs) were maintained in Dulbecco’s Modified Eagle’s Medium (DMEM) (Sial, Rome, Italy), supplemented with 10% heat-inactivated fetal bovine serum (iFBS) (Capricorn Scientific, Ebsdorfergrund, Germany), Gentamicin (50 mg/mL) (Bio Whittaker, Verviers, Belgium), Streptamycin (2 mg/mL) (Sial) and L-glutamine (2 mM) (EuroClone, Milan, Italy). Before the experiments, the cells were seeded in a 24-well plate (Corning, Glendale, AZ, USA) (5 × 10^5^ cells/well/mL) in DMEM plus 10% iFBS and then incubated for 24 h at 37 °C, with 5% CO_2_ to promote monolayer generation.

### 2.3. Antibodies

The monoclonal antibodies mouse anti-human complement component-5 (C5) (clone 10B6), mouse anti-human C5a/C5a des-arg (clone 2042) and mouse anti-human C3b/iC3b/C3d (clone 1H8) were kindly provided by Hycult Biotech (Uden, The Netherlands). The polyclonal antibody, goat anti-human complement component-3 (C3), was purchased from BioRad (Hercules, CA, USA). The monoclonal anti-human C3a/C3a des/arg antibody (clone K13/16) was obtained from MilliporeSigma (Merck KGaA, Darmstadt, Germany). The antibodies anti-C5 and anti-C3 were labeled using an FITC-Conjugation Kit (Abcam, Cambridge, UK). The antibodies anti-C5a/C5a des-arg, anti-C3b/iC3b/C3d and anti-C3a/C3a des/arg were labeled by an APC-Conjugation Kit (Abcam). The anti-C5aR1 antibody was purchased from Biolegend (San Diego, CA, USA). The anti-C3aR antibody was obtained from Miltenyi Biotech (Bergisch Gladbach, Germany).

### 2.4. Flow Cytometry Analysis

Flow cytometry analysis was performed to analyze the expression of the proteins C3, C5, C5a, C3a and C3b and the receptors C5aR1 and C3aR within VECs. The target proteins were detected in different flow cytometry panels. The VECs (5 × 10^5^ cells/well/mL) were incubated in DMEM + 10% iFBS with Colonizing and VVC strains (MOI 1:10) for 4 h at 37 °C, with 5% CO_2_. The cells were then washed with PBS to remove unbound *C. albicans*, harvested and centrifuged to remove the residual fungi in the culture. Next, the cells were incubated with the Fixable Viability Dye eFluor 780 (1:6000) (Invitrogen, Waltham, MA, USA) and fixed by Fixation Buffer (Biolegend) for 20 min in the dark at Room Temperature (RT). Subsequently, the cells were permeabilized with True-Phos Perm Wash (Biolegend) for 20 min at RT, and then incubated, for each flow cytometry measurement, in Perm Wash buffer including anti-human C5 (1:250), anti-human C5a/C5a des-arg (1:250), anti-human C3 (1:250), anti-human C3a/C3a des-arg (1:250), anti-human C3b/iC3b/C3d (1:250), anti-human C5aR1 (1:100) and anti-human C3aR (1:100) for an additional 20 min at RT in the dark. The cells were then washed and resuspended in PBS and recorded by FACSsymphony A1 (Becton Dickinson, Franklin Lakes, NJ, USA). In order to exclude the presence of C5aR1 and C3aR on the cell membrane, in selected experiments, the above-described staining procedures were performed without a permeabilization step. The data analysis was carried out by means of FlowJo_v10.10.0 software (Becton Dickinson); in each measurement, 8000 events corresponding to single and living cells were included in the analysis. Due to the non-specific binding observed in intracellular staining using isotype controls, we alternated FMO (Fluorescence Minus One) controls or isotype antibody controls to identify the correct gating strategy for the detection of true positive populations. With this approach, we minimized artifacts and ensured the accurate identification of positively labeled cells.

### 2.5. ELISA Test for the Detection of C3a and C5a in the Supernatant

The C3a and C5a levels were measured in cell culture supernatants from VECs stimulated with either the Colonizing or VVC strain, as well as those from unstimulated cells. Supernatants were collected after 24 h and centrifuged to remove all cells and cell debris. For the detection of C3a and C5a, the BD OptEIA Human C3a ELISA Kit and BD OptEIA Human C5a ELISA kit were employed, respectively (BD Biosciences, Franklin Lakes, NJ, USA), in accordance with the Manufacturer’s instructions, published elsewhere [31].

### 2.6. RNA Sequencing and Analysis

The RNA-seq analysis was performed as described by Sala et al. [6] and using already-published datasets. Briefly, VEC monolayers were infected with either the Colonizing or VVC strain (MOI 1:1), as described above. Cell pellets were sent for RNA preparation and NGS analysis using Illumina paired-end sequencing at Eurofins (Ebersberg, Germany) [32,33,34,35,36,37,38]. Gene expression data and sequence data are accessible in the NCBI Gene Expression Omnibus under accession number GSE207081. DEseq2 comparison of mRNA expression in VECs infected with Ca 14314 versus with Ca 01887 was used to determine the differential expression of cathepsin-family genes. DESeq2 is a powerful tool for the comparison of RNAseq data [38], and it was used through the Bioconductor package, as described in more depth previously [6].

### 2.7. Cathepsin D Activity Analysis

Cathepsin D activity within VECs, infected with the Colonizing or VVC strain, was evaluated by the Cathepsin D Activity Assay Kit (Fluorometric) (Abcam), following the Manufacturer’s instructions. Briefly, VECs (5 × 10^5^ cells/well/mL) were incubated with the Colonizing or VVC strain in a ratio of 1:10 (VECs: *C. albicans*) for 4 h or 24 h at 37 °C, with 5% CO_2_, in DMEM + 10% iFBS. After incubation, the cells were harvested and washed with PBS to remove *C. albicans*. Next, the cells were lysed by lysis buffer, and the lysates were incubated for 2 h at 37 °C in the dark with a reaction buffer containing Cathepsin D substrate. The fluorescence emission, corresponding to the Cathepsin D activity, was recorded by means of a microplate reader Fluoroscan FL (Thermo Fisher Scientific, Waltham, MA, USA) using an Ex/Em 328/460 nm filter. The data were analyzed by SkanIt software 5.0 (Thermo Fisher Scientific) and are expressed as a percentage (%) of activity, as compared to uninfected VECs.

### 2.8. Statistical Analysis

Statistical analyses were carried out using GraphPad Prism 10 software (GraphPad, Boston, MA, USA). The Shapiro–Wilk test was used to analyze data distribution within each experimental group. Differences between experimental groups were analyzed by a one-way ANOVA test followed by the uncorrected Fisher’s LSD multiple comparison test or by the Kruskal–Wallis test followed by the uncorrected Dunn’s multiple comparison test. Cathepsin D activity was analyzed by a paired Student’s *t*-test. Significance throughout the figures is indicated as follows: ns—not significant; significant increment—* *p* ≤ 0.05, ** *p* < 0.01, *** *p* <0.001 and **** *p* <0.0001; and significant reduction—# *p* ≤ 0.05, ### *p* < 0.001 and #### *p* < 0.0001.

## 3. Results

A monolayer of VECs was incubated with a cell culture medium or with the Colonizing or VVC *C. albicans* strain for 4 h at 37 °C with 5% CO_2_. Analysis of the complosome was performed by flow cytometry. The gating strategy was set to analyze only VECs, removing interference from *C. albicans* and considering only living single cells (Figure S1).

**Figure 1:**
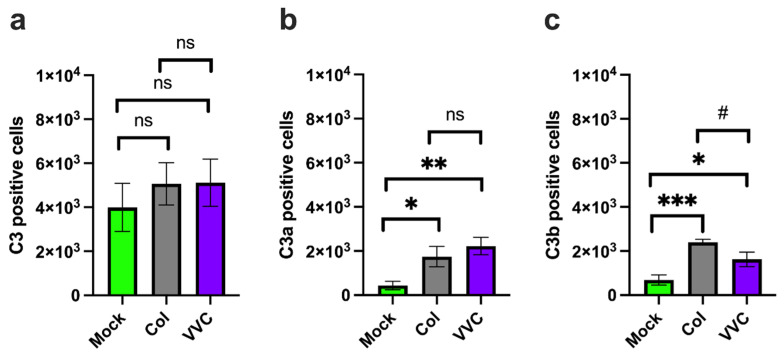
**C3, C3a and C3b analysis.** A monolayer of VECs was infected or not (Mock) with the Colonizing strain (Col) or the VVC strain (VVC) for 4 h. After incubation, levels of C3 (**a**), C3a (**b**) and C3b (**c**) within VECs were evaluated by cytofluorimetric analysis. Data in the graphs show the mean ± SEM of C3-, C3a- and C3b-positive cells (cell counts from 8000 live cells) obtained from 4 different experiments. ns—not significant; * *p* ≤ 0.05; # *p* ≤ 0.05; ** *p* < 0.01; *** *p* < 0.001.

First, we analyzed the presence of C3 and its cleavage fractions C3a and C3b within VECs. Our results show that approximately half of the VECs analyzed were positive for C3 (49.8%), with a non-significant increase after infection with either the Colonizing (63.2%) or VVC (63.8%) strain (Figure 1a). The C3a-positive VEC cell fraction was low in uninfected cells (5.4%), and this fraction significantly increased after infection with both Colonizing (21.7%) and VVC (27.7%) strains. Interestingly, no differences in the fraction of C3a-positive infected cells were observed relative to the strain used for the infection (Figure 1b). Similarly, the fraction of C3b-positive VECs was low in uninfected cells (8.5%) and significantly increased after infection with both the Colonizing (29.9%) and VVC (20.3%) strains. However, a significantly lower fraction of C3b-positive VECs was detected in VECs infected with the VVC strain as compared to those infected with the Colonizing strain (Figure 1c).

**Figure 2:**
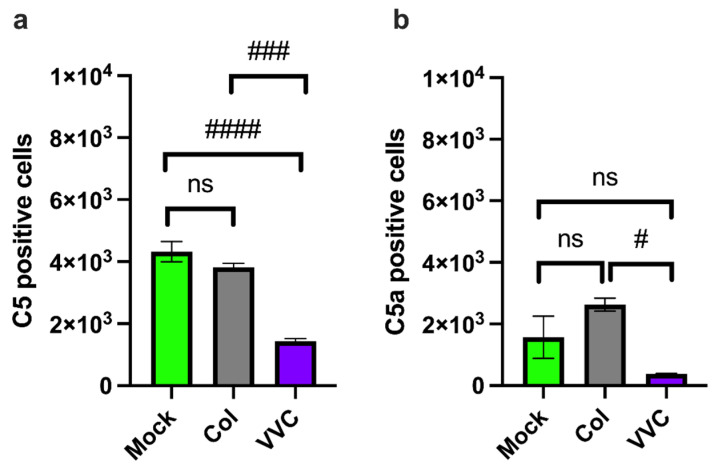
**C5 and C5a analysis.** A monolayer of VECs was infected or not (Mock) with the Colonizing strain (Col) or the VVC strain (VVC) for 4 h. After incubation, the levels of C5 (**a**) and C5a (**b**) within VECs were evaluated by cytofluorimetric analysis. Data in the graphs show the mean ± SEM of C5- and C5a-positive cells (cell counts from 8000 live cells) obtained from 3 different experiments. ns—not significant; # *p* ≤ 0.05; ### *p* < 0.001; #### *p* < 0.0001.

Next, we measured C5 and C5a within VECs. Here, approximately half of the VECs analyzed were positive for C5 (54%), and this level was maintained upon infection with the Colonizing strain (47.6%). However, the amount of C5-positive VECs was significantly reduced upon infection with the VVC strain (17.9%), as compared to uninfected or Colonizing strain-infected VECs (Figure 2a). In parallel, a significantly lower number of C5a-positive VECs were observed upon infection with the VVC strain, as compared to infection with the Colonizing strain (Figure 2b).

**Figure 3:**
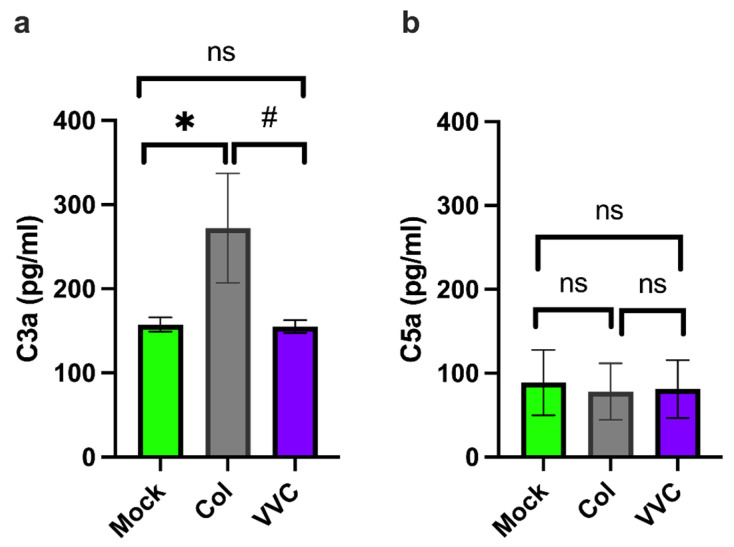
**Extracellular release of C3a and C5a.** A monolayer of VECs was infected or not (Mock) with the Colonizing strain (Col) or VVC strain (VVC) for 24 h. After incubation, supernatants were collected and tested for the extracellular release of C3a (**a**) and C5a (**b**) by a specific ELISA kit. Data in the graphs show the mean ± SEM of extracellular C3a and C5a from 5 different experiments. ns—not significant; * *p* ≤ 0.05; # *p* ≤ 0.05.

An ELISA assay was performed on the supernatants from VECs uninfected or infected for 24 h with the Colonizing or VVC strain to assess the C3a and C5a release upon infection. The results showed a significant increase in the release of the anaphylatoxin C3a from VECs infected with the Colonizing strain compared to uninfected cells or those infected with the VVC strain. The analysis did not show any difference between uninfected VECs or VVC strain-infected VECs (Figure 3a). No differences in C5a release were observed in the context of infection either (Figure 3b).

**Figure 4:**
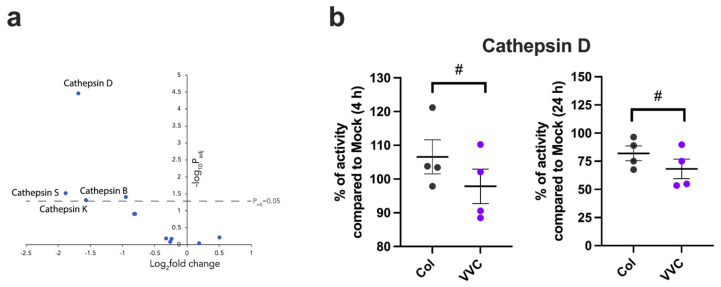
**RNAseq analysis of cathepsin genes and Cathepsin D activity.** (**a**) Volcano plot of the log_2_ ratio of gene expression between the VVC strain and the Colonizing strain (x-axis) against the −log_10_-transformed adjusted *p*-value, as calculated in DESeq2, for each cathepsin gene expressed above background (Cathepsins A, B, C, D, F, H, K, L, S, V and Z). (**b**) Cathepsin D activity in VECs infected with the Colonizing strain (Col) or the VVC strain (VVC) after 4 h or 24 h. Data in the graphs in (**b**) show the mean ± SEM of the % of Cathepsin D activity in infected compared to uninfected VECs, obtained from 4 different experiments. # *p* ≤ 0.05.

To explain the tendency of the Colonizing strain to induce greater cleavage of C3 and C5 in VECs upon infection, RNAseq analysis of an already-published dataset was performed [6]. The analysis was conducted on VECs infected with either the Colonizing or VVC strain. We focused on the cathepsin family, which is known to participate in the cleavage of intracellular complement components [14]. We found an overall trend toward the down-regulation of the whole cathepsin family, with significantly lower gene expression of Cathepsins B, D, K and S in VECs infected with the VVC strain compared to those infected with the Colonizing strain (Figure 4a). We then tested whether lower cathepsin expression is correlated with lower activity; in this context, Cathepsin D activity was directly measured in infected VECs [27]. Consistent with the gene expression results, we found a significant reduction in Cathepsin D activity in VECs infected with the VVC strain compared to cells infected with the Colonizing strain after 4 h and 24 h (Figure 4b).

**Figure 5:**
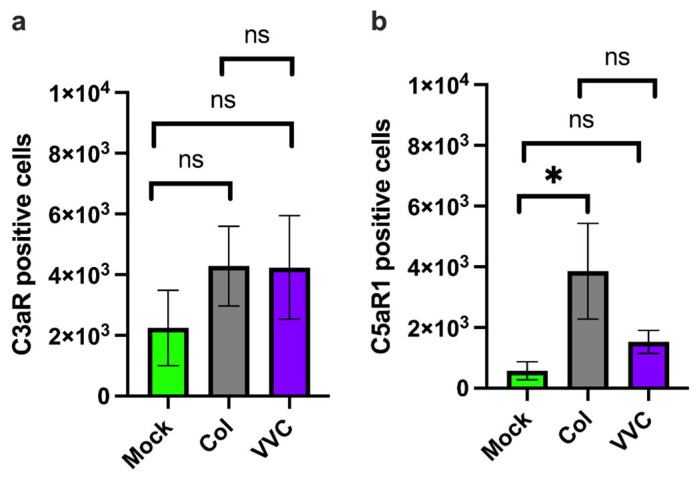
**Intracellular C3aR and C5aR1 analysis.** A monolayer of VECs was infected or not (Mock) with the Colonizing strain (Col) or VVC strain (VVC) for 4 h. After incubation, intracellular C3aR (**a**) and C5aR1 (**b**) were analyzed by cytofluorimetric analysis. Data in the graphs show the mean ± SEM of C3aR- and C5aR1-positive cells (cell counts from 8000 live cells) obtained from 4 different experiments. ns—not significant; * *p* ≤ 0.05.

The presence of cell-associated C3a, C3b, C5a and C5b suggests that there is activation of the complosome in infected VECs. To determine if the VECs can respond to elevated levels of intracellularly produced complement components, we also analyzed the expression and potential modulation of C3a and C5a intracellular receptors: C3aR and C5aR1. Our results show that the number of C3aR-positive VECs did not significantly increase upon infection with either the Colonizing or VVC strain, as compared to uninfected VECs (Figure 5a). In contrast, a significant increase in C5aR1 expression was observed after infection with the Colonizing strain but not with the VVC strain (Figure 5b). No VECs positive for membrane-bound C3aR and C5aR1 were found.

## 4. Discussion

While the complement system clearly plays important roles in anti-*Candida* immunity in systemic infection, it is still largely unknown whether it protects against mucosal disease. Recent work suggests that intracellular activation of complement in the “complosome” can change cellular physiology, but the role of the complosome in vaginal epithelial cells (VECs) has not yet been characterized. Our results highlight cell-associated complement modulation in VECs by *C. albicans* infection in vitro, with distinct immune responses prompted by Colonizing and VVC strains. By setting off more effective cleavage of intracellular complement components, the Colonizing strain may help VECs protect themselves against invasion, damage and exfoliation. This could establish a mutual interaction that provides reciprocal advantages to both the host and microbe. In contrast, the VVC strain down-regulates or prevents aspects of intracellular activation of C3 and C5, in concert with a reduction in cathepsin expression and activity. This dysregulation of the complement response in VECs could help the VVC strain evade protective epithelial immune mechanisms and limit immune cell recruitment, ultimately promoting the progression from infection to disease.

In this comparative study, we have used a Colonizing strain and a VVC strain of *C. albicans* that had been previously characterized [5] and employed in an in vitro infection model of VECs. These strains differentially activate specific epithelial pathways (such as the type I IFN pathway), fungal shedding and epithelial exfoliation. The differential activation of the complosome by the Colonizing vs. VVC strain is consistent with the enhanced ability of the VVC strain to cause epithelial exfoliation and block type I IFN activation [6].

Complement Factor 3 (C3) is a crucial mediator of the entire complement cascade. We find that C3 cleavage to produce C3a is induced by both *C. albicans* strains, but only the Colonizing strain stimulates extracellular secretion of C3a [39]. In turn, the production of C3b is also induced by both strains but to a significantly greater extent by the Colonizing strain [39]. As C3a recruits neutrophils, this suggests that the Colonizing strain may attract stronger recruitment to the vagina through this pathway. Since C3b is crucial for opsonization and fungal killing [9], this result could indicate that VECs retain their ability to counteract intracellular fungal invasion upon infection with a Colonizing strain, whereas such capacity might be reduced upon infection with the VVC strain. There is an intriguing disparity in the frequency of C3a- and C3b-positive cells, which could be due to selective degradation of C3b by proteases produced by or elicited by the VVC strain.

Complement Factor 5 (C5), which is cleaved to generate the anaphylatoxin C5a, is present and processed at basal levels during VEC infection with the Colonizing strain but is significantly reduced following VVC-strain infection. This result mirrors the observed down-regulation in gene expression of several members of the cathepsin family, particularly Cathepsin D but also Cathepsins A, B, C, F, H, K, L, S, V and Z, in VVC-infected VECs. Moreover, Cathepsin D activity was also markedly reduced. Since cathepsins mediate intracellular C3 and C5 cleavage, our data suggest that the VVC strain impairs C5 cleavage through the down-regulation of cathepsin activity [27]. The lack of C5a in VEC supernatants suggests a greater involvement of C5a in the intracellular compartment, contrasting with the potential role of extracellular C3a as an anaphylatoxin in promoting immune cell recruitment in response to fungal infection [40].

**Figure 6:**
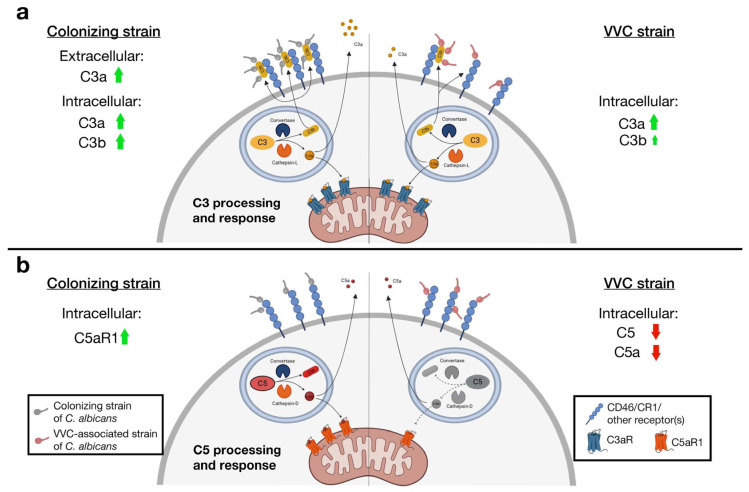
**Activation of complosome in VECs by *C. albicans*.** Differential activation of C3, C3a and C3b (**a**) and C5 and C5a (**b**) and intracellular C3aR and C5aR1 involvement in VECs infected with the Colonizing or VVC-associated strain. Arrows indicate changes in the levels of complement factors and receptors after infection with either the Colonizing strain (left) or the VVC strain (right). Green indicates increased levels and red indicates lower levels as compared to resting levels. Created with BioRender (adapted from King B.C. and Blom A.M., 2024 [39]).

Of the key complement factor receptors, only the Colonizing strain significantly increased intracellular C5aR1, while intracellular C3aR was unchanged in all infections. These results strengthen our idea that the Colonizing strain is more prone to stimulate the complosome and that the C5a/C5aR1 and C3a/C3aR axes seem to be partly compromised in VECs infected with the VVC strain. Figure 6 summarizes our hypothesis of complosome modulation in VECs by the Colonizing and VVC-associated strains.

In fungal infections, components of the fungal cell wall such as β-glucans and galactomannans have been reported to activate complement [41]. Nonetheless, several evasion strategies have been adopted by fungal cells to avoid complement, such as binding to the Factor H complement inhibitor and the secretion of proteases (such as Secreted Aspartyl Proteases by *C. albicans*) that cleave complement components, thus impairing complement function [41,42].

One important question that these results bring up is how different *C. albicans* strains might elicit divergent VEC complosome responses. Several virulence factors have previously been linked to complement regulation: β-glucan masking blocks complement components impairing the lectin pathway [43,44]; the secretion of Pra-1 and Secreted Aspartyl Proteases (Saps) enables *C. albicans* to degrade the complement proteins C3b, C4b and C5, thus inhibiting the formation of MAC and blocking the classical and alternative pathways [9,44]; and specific sequence variations in *C. albicans* Sap2p (such as the V273L substitution) increase the degradation of C3 and C3b, thus reducing complement activation [45]. Thus, the VVC strain may have greater Sap activity and β-glucan masking, thus limiting complosome activation and enhancing its ability to cause VEC damage. There is an open question as to the overall ability of diverse vaginal *C. albicans* strains to activate the complosome. We have only tested one Colonizing and one VVC strain here, and it is yet to be determined if all or most Colonizing strains behave like Ca 14314 and strongly activate the complosome, where most or all VVC strains act like Ca 01887 and provoke significant complosome inactivation. The testing of diverse clinical isolates from healthy patients and those with disease will be crucial in answering this question.

Another important question elicited by these findings is whether complosome activation is required for VECs to resist *C. albicans* invasion, damage and exfoliation. Given that intracellular complement receptors are located on mitochondria [46], their activation may regulate mitochondrial function, leading to a different activation of the type I IFN pathway through several mechanisms, such as a different release of mtDNA or ROS production. Protective type I interferon pathway activation has been suggested to be provoked in VECs through mitochondrial depolarization, leading to mtDNA release and likely to the activation of the cGAS-STING pathway [47]. Our recent work links type I interferon activation to Colonizing strains, where the blockade of the type I interferon receptor enhances fungal shedding in VEC challenges [6]. Taken together, these results suggest a potential mechanism for differential damage and exfoliation caused by the VVC and Colonizing strains used here, where only the Colonizing strain activates the complosome vigorously, exacerbating mitochondrial disruption and IFN-I activation, thereby protecting VECs from infection.

## 5. Conclusions

In sum, we have demonstrated that *C. albicans* can stimulate VEC complosome activation in a strain-specific manner. These experiments identify a new physiological response to *C. albicans* and raise important questions as to the physiological consequences of complosome activation (or lack thereof) in VECs, and its role(s) in asymptomatic colonization and symptomatic VVC.

This raises the possibility that targeting the complement system, for example, through the modulation of the C5a/C5aR1 and/or C3a/C3aR axes, may provide an effective novel approach to counteract fungal infection of the vaginal mucosa.

More studies are needed to further elucidate the tissue-specific immune regulatory functions of complement and the contribution of complosome activation in mucosal fungal diseases. These studies should include an analysis of how intracellular C3aR and C5aR1 activation regulates *C. albicans* adhesion to and invasion of the epithelium, modifies epithelial integrity and modulates epithelial functions relevant to fungal infection.

From a clinical perspective, these immune differences in response to VVC and Colonizing strains offer promising avenues for improved diagnosis and therapeutic strategies. For instance, levels of epithelial C5a or cathepsin activity could serve as biomarkers in vaginal specimens that could help distinguish asymptomatic colonization from patients with VVC/RVVC. Alternatively, acute upregulation of complosome activation through drugs that turn on cathepsin activity might counteract the limited complosome activation provoked by VVC strains and thus serve as an immunomodulatory treatment for VVC.

## Data Availability

The original contributions presented in the study are included in the article. Further inquiries can be directed to the corresponding authors.

## Supplementary Materials

The following supporting information can be downloaded at https://www.mdpi.com/article/10.3390/jof11070501/s1, Figure S1: Representative gating strategy applied for VEC discrimination from *C. albicans*.
