# Supplementary material for: Vaginal Clinical Isolates of Candida albicans Differentially Modulate Complosome Activation in Vaginal Epithelial Cells
Source: J Fungi (Basel). 2025 Jul 3;11(7):501. doi: 10.3390/jof11070501 (PMC12296101; doi:10.3390/jof11070501)
Supplement: Supplementary file 1 [file jof-11-00501-s001.zip › jof-3717290-supplementary.pdf]

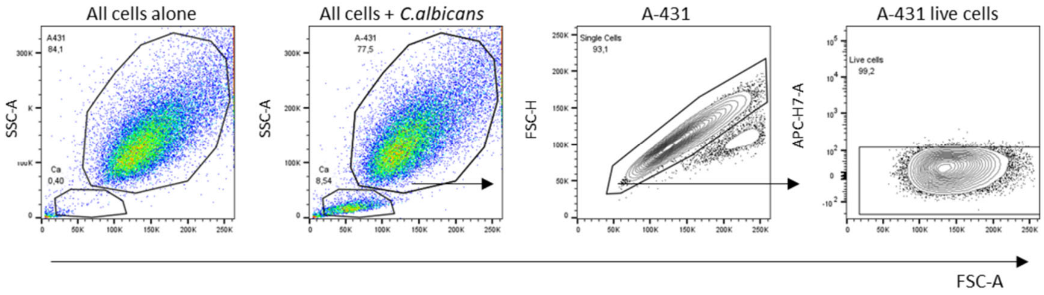

Figure S1. Representative gating strategy applied for VECs discrimination from *C. albicans*. In all experiments for flow cytometry, VECs were incubated alone or with Colonizing or VVC strains for 4 hr at 37°C, with 5% of CO_2. After infection, the cells were harvested, washed, permeabilized and then labelled for specific complement components. The cells were discriminated from interfering *C. albicans* and only VECs (single and living) were included in the statistical analysis.
